# In situ high temperature X-ray diffraction and dilatometric analysis of CGO–Cu composites for solid oxide devices

**DOI:** 10.1038/s41598-026-35161-w

**Published:** 2026-01-10

**Authors:** M. Balaguer, M. Fabuel, A. Kriele, A. Stark, J. M. Serra, C. Solís

**Affiliations:** 1 Instituto de Tecnología Química (ITQ), Consejo Superior de Investigaciones Científicas-Universitat Politècnica de València, 46022 Valencia, Spain; 2 German Engineering Materials Science Centre (GEMS) at Heinz Maier-Leibnitz Zentrum (MLZ), Helmholtz-Zentrum Hereon, 85748 Garching, Germany; 3 Institute of Materials Physics, Helmholtz-Zentrum Hereon, Max-Planck-Strasse 1, 21502 Geesthacht, Germany

**Keywords:** CGO−Cu cermets, Composites, Thermal expansion, In situ synchrotron XRD, Solid oxide cells, Energy science and technology, Materials science

## Abstract

Understanding the thermo-mechanical compatibility of composite electrodes is essential for the long-term reliability of solid-oxide electrochemical devices. In this study, we demonstrate a combined in situ synchrotron X-ray diffraction (XRD) and simultaneous dilatometry approach as a rapid and predictive method to quantify both phase-resolved and bulk thermal expansion while tracking microstructural evolution at operational temperatures. Ce_0.8_Gd_0.2_O_2−δ_–Cu (CGO–Cu) composites with varying CGO: Cu ratios (39:61–70:30 vol%) were synthesized as potential anode materials compatible with CGO electrolytes up to 800 °C. In situ XRD confirmed only the CGO and Cu phases, with Rietveld refinement revealing a slight lattice expansion and reduced CGO crystallite size with increasing CGO content. Concurrent dilatometry indicated systematic changes in the macroscopic thermal expansion and densification behavior, which correlated with the phase and microstructural evolution observed during heating. The CGO–Cu (59:41) composite exhibited a nearly temperature-independent coefficient of thermal expansion consistent with the rule-of-mixtures predictions and minimal high-temperature shrinkage. These findings validate the combined in situ synchrotron XRD + dilatometry methodology as a powerful approach for characterizing and capturing the TEC characteristics of cermets, and for guiding the design of thermomechanically compatible oxide-metal composites for high temperature electrochemical applications.

**Supplementary Information:**

The online version contains supplementary material available at 10.1038/s41598-026-35161-w.

## Introduction

The long-term stability of solid oxide fuel cells (SOFC) and electrolysis cells (SOEC), and catalytic membrane reactors, is governed not only by their electrochemical performance but also by the thermal and mechanical compatibility of their constituent components, particularly the electrolyte and electrodes^1,2^. Thermal‑expansion coefficient (TEC) mismatch, chemical reactivity, and insufficient mechanical integrity at high temperatures can induce interfacial degradation, delamination, and cracking, ultimately limiting the efficiency and lifetime of these electrochemical devices. Therefore, ensuring similar thermal expansion behaviors across these materials is critical for maintaining mechanical integrity and manufacturability. These devices operate with high efficiency at elevated temperatures, and these conditions, along with thermal cycling, can induce mechanical stress and accelerate material degradation. Specifically, electrode oxidation resulting from fuel and steam separation limits both the operational stability and device lifetime. Consequently, lowering the operating temperature has become a key strategy for enhancing the long-term performance and reliability. Conventional solid-oxide cells typically employ an yttria-stabilized zirconia (YSZ) electrolyte between a porous Ni/YSZ anode and a porous lanthanum-strontium manganite (LSM) cathode, operating at temperatures above 800 °C^3,4^. To enable the operation of these devices at lower temperatures, the development of advanced electrolyte materials is essential. One promising candidate is Ce_0.8_Gd_0.2_O_2−δ_ (CGO), an oxygen-ion conductor that exhibits high ionic conductivity in the intermediate temperature range of 600–800 °C. Although CGO develops mixed ionic–electronic conductivity under reducing conditions, which can cause electronic leakage, the use of thin CGO electrolytes allows a substantial reduction in ohmic losses. This approach can partially compensate for the intrinsic limitations of CGO and enable competitive performance at intermediate temperatures, albeit without fully eliminating electronic leakage effects^5,6^. Therefore, it is crucial to develop compatible anode materials exhibiting sufficient electronic conductivity and electrocatalytic activity while also providing the mechanical robustness necessary to support thin CGO electrolytes^7^.

Among the various anode materials, Cu-based ceria cermets have demonstrated significant advantages over conventional Ni-based cermets for the direct oxidation of hydrocarbons. This is primarily because they are less prone to carbon coking, which is a common degradation mechanism in Ni-containing materials. In these cermets, Cu provides the necessary electronic conductivity, whereas a doped ceria contributes to the oxide-ion transport and the catalytic activity of the oxidation reactions, partially compensating for the catalytic role typically fulfilled by Ni in standard Ni-YSZ electrodes. Moreover, Cu is a more economically viable alternative to Ni, further enhancing its suitability for large-scale and cost-effective applications^7–9^.

To ensure the mechanical robustness of these electrochemical devices at high temperatures, all constituent materials should exhibit similar matched thermal expansion coefficients (TEC). Mismatches in the TEC can induce thermal stress during operation and thermal cycling, leading to mechanical degradation. Notably, even a deviation as small as 7 × 10^− 6^ K^− 1^ between the TEC of the electrode and electrolyte has been shown to significantly degrade fuel cell performance, primarily due to electrode delamination^7^. This work combines in situ high‑energy synchrotron X-ray diffraction (XRD) with simultaneous dilatometry to study CGO–Cu cermets with varying CGO: Cu ratios (39:61 to 70:30, vol%). The principal targets of this study are as follows: (i) to quantify phase‑specific lattice expansion and apparent crystallite sizes; (ii) to assess bulk thermal expansion; (iii) to relate microstructure to macro‑scale TEC; and (iv) to identify compositions with the most stable thermo‑mechanical behavior versus temperature.

## Experimental

### Powder synthesis and bar fabrication

CGO–CuO composite powders were prepared via co-precipitation. Aqueous solutions of CeN_3_O_9_·6H_2_O 99%, CuN_2_O_6_·2.5H_2_O 98% and GdN_3_O_9_·6H_2_O 99.9% (Sigma Aldrich) were mixed at 60 °C in the target cation ratios. A 0.75 M (NH_4_)_2_CO_3_ solution was added dropwise to induce precipitation, followed by the slow addition of 2 M NaOH to pH = 12. The suspension was stirred at room temperature (RT) for 24 h, filtered, washed with deionized water, dried at 100 °C overnight, and calcined in air at 800 °C.

The powders were ball-milled with acetone using zirconia media for 16 h, dried, sieved, and uniaxially pressed into bars (ca. 10 × 4 × 2 mm) at 20 kN. Six bar samples with different CGO–CuO ratios ranging from 40:60 to 70:30 vol% were prepared and sintered at 1050 °C (10 h). Then, they were reduced to CGO–Cu in 5% H_2_/N_2_ at 800 °C (10 h) to form the cermets.

### Densification, microstructure, and composition

The bulk density was measured using the Archimedes method. Morphological characterization was performed using a ThermoFisher Scientific Quattro S field emission environmental scanning electron microscope (ESEM) operated by the Helmhotz-Zentrum Hereon and Jülich Center for Neutron Science (JCNS). The SEM micrographs were taken at a working distance of 10 mm with a probe current of 238 pA and an acceleration voltage of 10 kV using an on-lens Si p-n diode circular backscatter detector (CBS) to increase the material contrast. The particle sizes were measured using the ImageJ open software^10^. Energy dispersive X-ray spectroscopy (EDS) was performed with a ThermoFisher EDS UltraDry Si-drift detector (60 mm²) at a take off angle of 40° with an energy resolution of 127 eV Mn Kα at an e-beam emission current of 500 pA. An acceleration voltage of 15 kV and a probe current of 1.2 nA were used resulting in a count rate of 21 kcps.

### In situ synchrotron XRD and simultaneous dilatometry

**Fig. 1:**
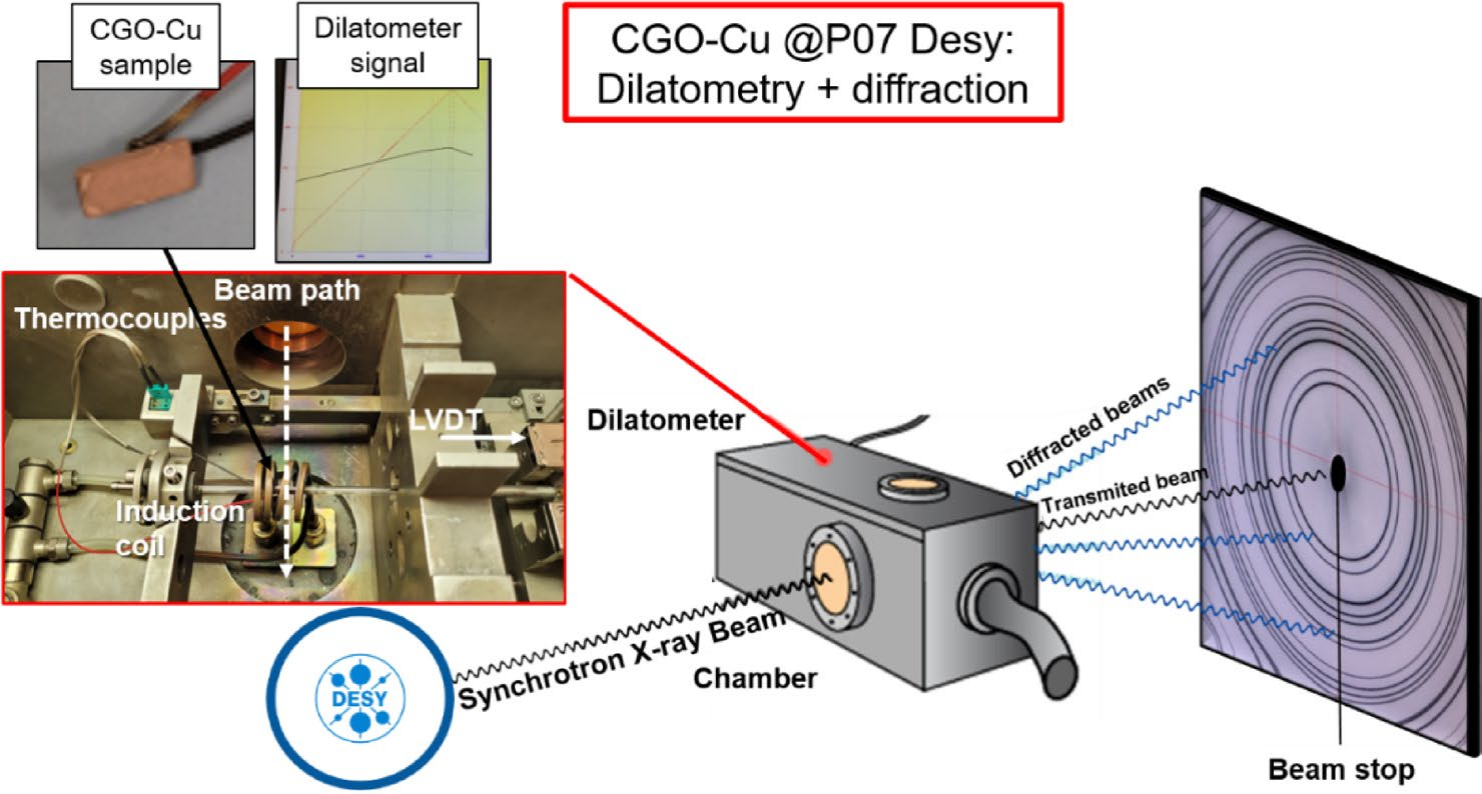
Sketch of the in-situ XRD and dilatometer setup used at Desy. A picture of a CGO: Cu bar sample, the thermal expansion recorded by the dilatometer. The interior of the dilatometer and the diffracted rings are also shown.

Synchrotron X-ray diffraction (XRD) patterns were obtained at the high-energy materials science (HEMS) beamline P07^11^ at the DESY synchrotron facility in Hamburg, Germany (Deutsches Elektronen-Synchrotron). The photon energy used was 87 keV (λ = 0.1425112 Å, and first harmonic as it was the side station). A modified Bähr DIL 805 A/D dilatometer was used to inductively heat the samples from 25 to 800 °C in vacuum at a rate of 10 °C/min, in order to record the change in length of the samples while performing the XRD measurements. A PerkinElmer XRD1621 image detector with a pixel size of 200 × 200 µm^2^ and a resolution of 2048 × 2048 pixels was used to record the XRD patterns. The detector was positioned with a sample-to-detector distance of approximately 1700 mm, ensuring that the primary beam hit the detector center, allowing for the recording of complete Debye-Scherrer rings. The experimental setup for the in situ XRD and dilatometer characterization can be seen in Fig. 1. A 2 mm LaB_6_ standard sample (NIST standard reference material SRM-660a) was used for the calibration. The 2D patterns from the synchrotron measurements were treated with the Fit2D software^12^. Finally, the Full-Prof package^13^ was used for the Rietveld refinement^14^ of the acquired synchrotron diffraction patterns.

### Thermal expansion analysis

The phase-resolved linear TEC from XRD was computed as:

1 $$\:{\alpha\:}_{XRD}=\frac{1}{{a}_{0}}\left(\frac{da}{dT}\right)$$

where *a* is the cubic lattice parameter.

Bulk dilatometric data were fitted using the measured thermal expansion:

2 $$\:\frac{{\Delta\:}L}{{L}_{1}}={c}_{1}+{c}_{2}T+{c}_{3}{T}^{2}+{c}_{4}{T}^{3}$$

considering the following relation

$$\:\mathrm{T}\mathrm{E}\mathrm{C}={\alpha\:}_{dil}=\frac{1}{{L}_{1}}\left(\frac{dL}{dT}\right)={c}_{2}+{2c}_{3}T+{3c}_{4}{T}^{2}$$

where *L*_*1*_ is the initial length of the sample and *ΔL* is the change in the length of the sample in a certain temperature range. *c*_*1*_, *c*_*2*_, *c*_*3*,_ and *c*_*4*_ are the experimental constants, and *T* is the temperature (K).

## Results and discussion

**Fig. 2:**
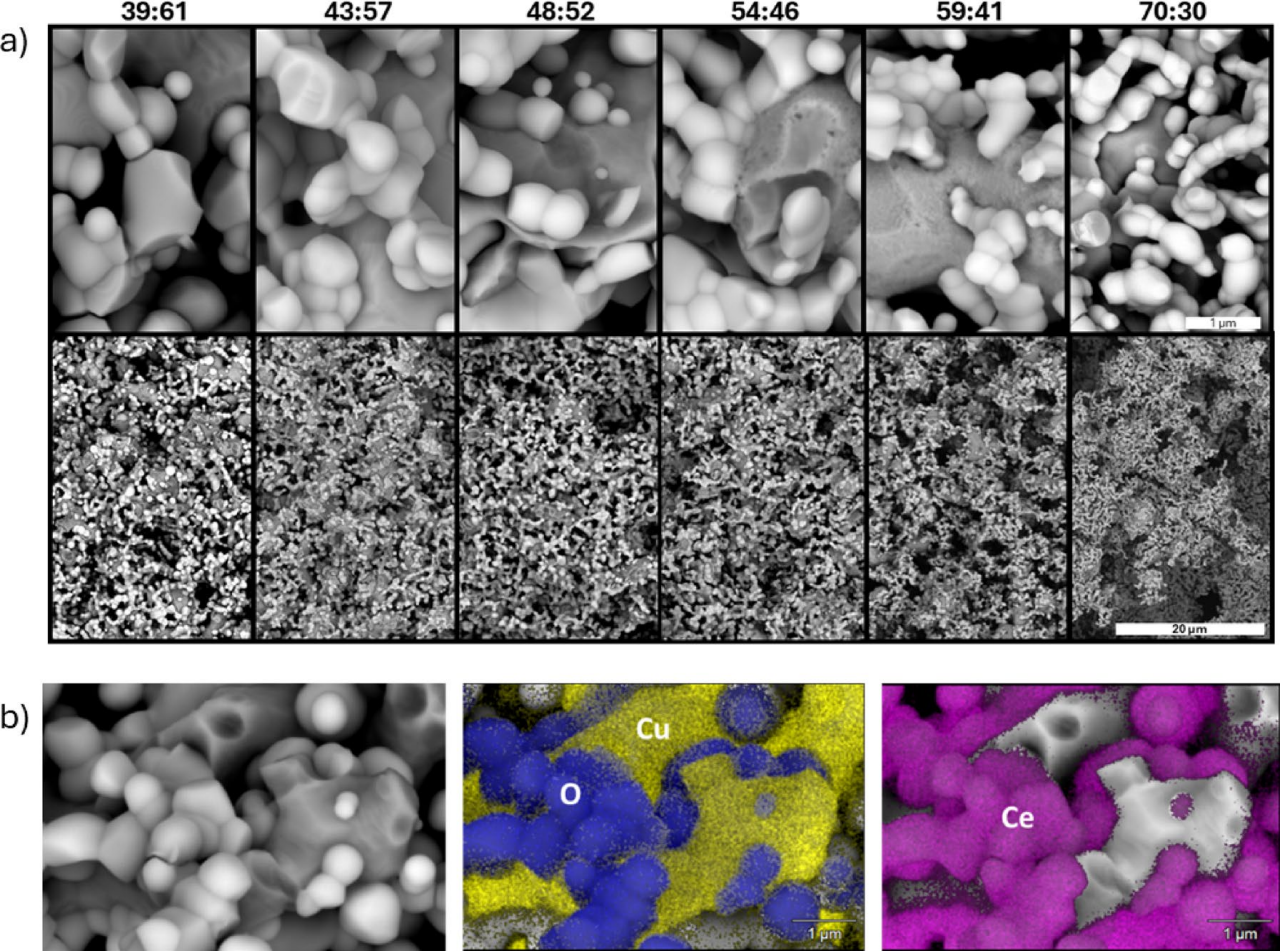
**a** SEM images of studied cermets at two different magnifications, and **b** EDS analysis of 39:61 CGO: Cu composite. Note that the ratios on top of the pictures correspond to the final CGO: Cu vol% ratios (Table 1).

Six compositions spanning 40:60 to 70:30 (CGO: CuO, vol%) were fabricated. Sintering at 1050 °C for 10 h typically yielded mechanically sound bars; shorter holds or lower temperatures often produced insufficient densification (and bars broke). The final reduction in 5% H₂ at 800 °C converted CuO to Cu (39:61 to 70:30 CGO: Cu vol% as will be shown later) and completed cermet formation. Accordingly, the morphologies and compositions of the different composites were analyzed using ESEM. The backscattered SEM images (Fig. 2a) reveal bright CGO and darker Cu regions resulting from the atomic number contrast. EDS confirmed the phase assignments for 39:61 CGO: Cu as an example (Fig. 2b).

**Fig. 3:**
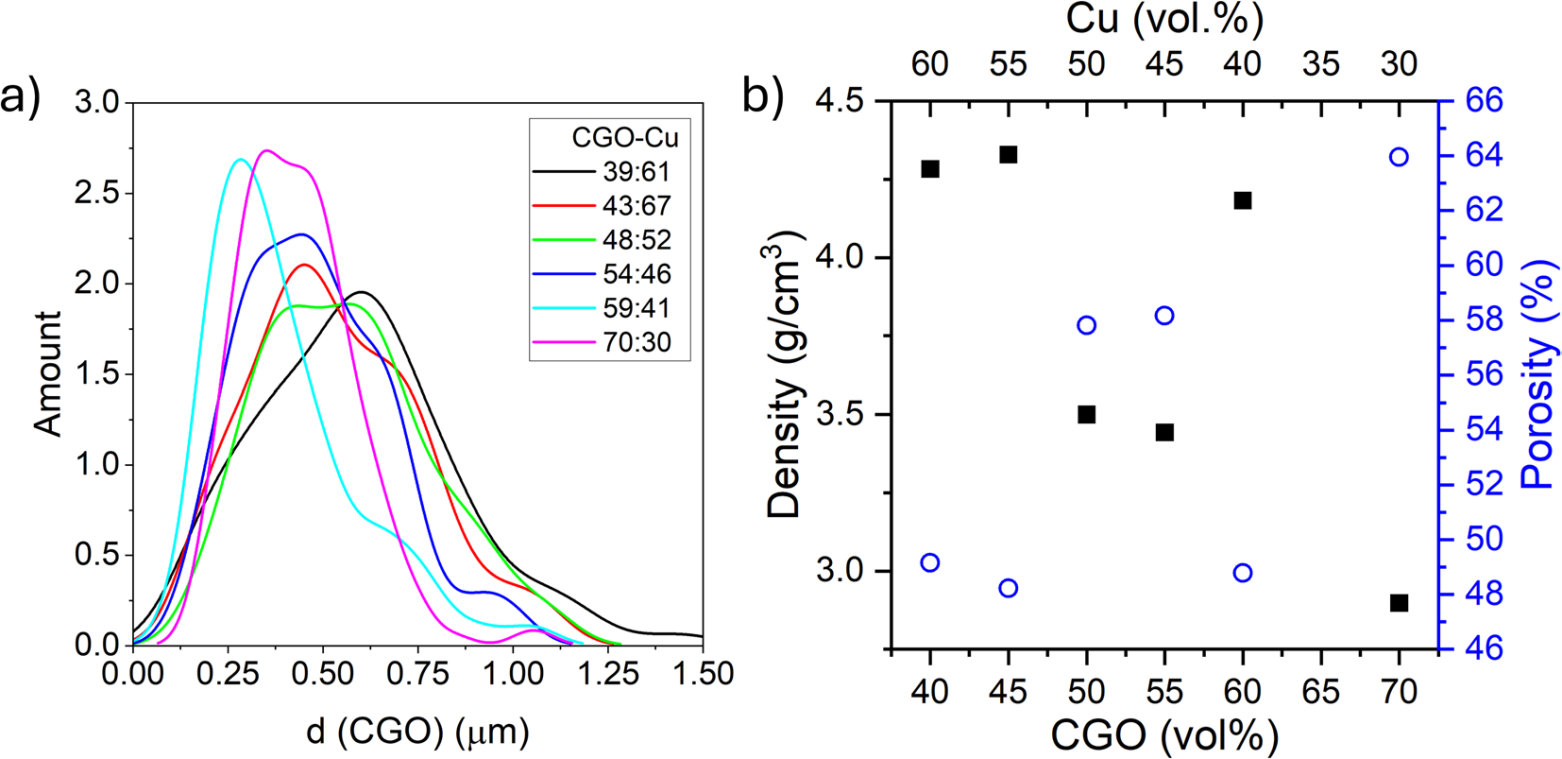
**a** Obtained CGO size distribution from SEM images and **b** density (left axis) and porosity (right axis) obtained from Archimedes method. Note that the ratios correspond to the final CGO: Cu ratios (Table 1).

Across all compositions, the Cu phase appeared highly melted and coalesced, forming a continuous or semi-continuous morphology in which individual particles or grains cannot be clearly distinguished or separated. This behavior is attributed to the high surface and grain-boundary diffusivity of Cu at reducing temperature (800 °C), combined with solid-state wetting and redistribution during the reduction of CuO to metallic Cu. These processes lead to the formation of finely dispersed and interconnected Cu regions along CGO surfaces and grain boundaries, rather than discrete, well-defined Cu particles. As a result, a direct measurement of particle or grain size from SEM images is not feasible. Differently, the CGO particle size slightly decreased as the CGO fraction increased, as quantified from the high‑magnification images (SEM images shown in Figure S1) and quantified in the size distributions summarized in Fig. 3a. Low‑magnification SEM (bottom of Fig. 2a) suggests lower porosity at higher Cu loadings, as corroborated by Archimedes. The density and corresponding calculated porosities of reduced samples are shown in Fig. 3b, left and right axis, respectively. The porosity values agree with literature^15^ and decrease from 64% to 48% with increasing Cu content, except for 59:41 sample, which is denser than expected. Reduction of CuO to Cu causes local volume contraction, leading to a marked increase in porosity compared to the as prepared CGO–CuO materials (8.5–13%, Supporting Figure S2). However, within the reduced composites, porosity decreases as Cu content increases. This trend is attributed to the high mobility of Cu at 800 °C, which promotes solid-state wetting and redistribution along CGO surfaces and grain boundaries^16,17^. The mobile Cu phase enhances capillary-driven particle rearrangement and pore filling, and at higher Cu loadings forms an interconnected network, resulting in densification despite the intrinsic CuO → Cu volume shrinkage.

**Fig. 4:**
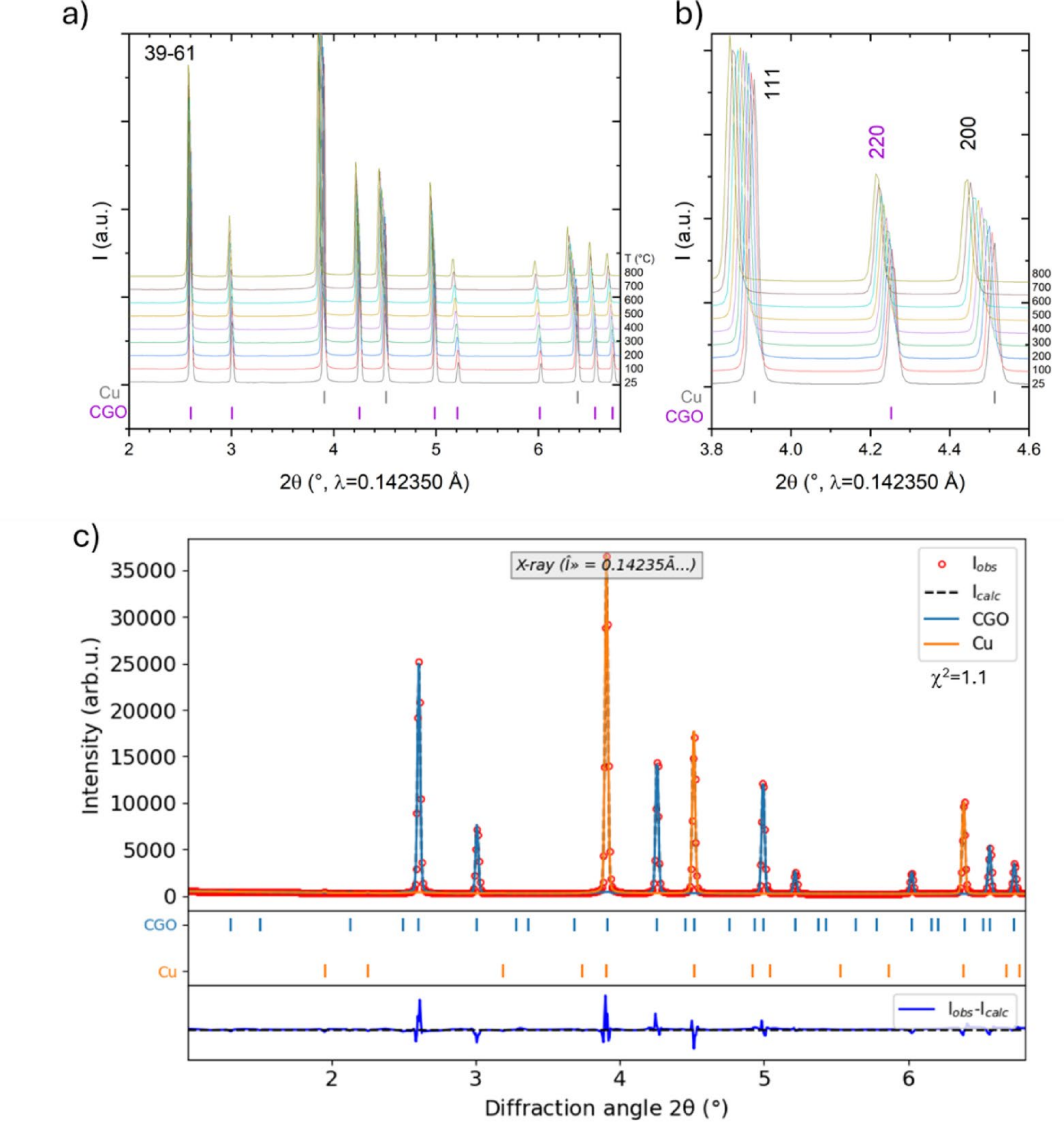
**a** XRD patterns of sample 39:61 from RT up to 800 °C, and **b** zoom between 2θ = 4.1° and 4.6 ° (also expected positions for Cu and CGO phases at the bottom); **c** Rietveld refinement of the sample 39:61 at RT (R_p_=2.61, χ^2^ = 1.1). The red circles correspond to measured data, the black-dashed line to the total calculated data (which is the sum of intensities from CGO and Cu phases), and the blue line to the difference between the observed and calculated intensities at each data point. Besides, the positions of the CGO and Cu Bragg reflections are depicted.

A detailed microstructural characterization of the composites and their high-temperature behavior was performed using in situ synchrotron XRD during dilatometer investigations. The XRD patterns for 39:61 (CGO: Cu) from RT to 800 °C (Fig. 4a) show only CGO (fluorite) and Cu (fcc) reflections with no secondary oxides during heating under vacuum. A magnified view of the XRD data for the 39:61 sample is shown in Fig. 4b, highlighting the gradual shift of the (200) Cu and (220) CGO reflections toward lower angles with increasing temperature, which is indicative of lattice expansion due to thermal effects. The expected Bragg reflection positions at RT for the Cu and CGO phases are also indicated (Fig. 3a and b).

XRD patterns were analyzed using Rietveld refinement, which identifies the phases present in each sample and determines their weight fractions (wt%) and lattice parameters. An example of the Rietveld refinement for the sample 39:61 at RT is depicted in Fig. 4c. Table 1 summarizes the weight%, lattice parameters and derived volume fractions for all compositions at RT, calculated using the densities derived from the Rietveld analysis.

**Table 1:** Nominal CGO: CuO sample composition (vol%), and the RT weight fractions and lattice parameters of the CGO and Cu phases obtained after reduction, along with the calculated vol% of each phase.

Nominal vol%	CGO @ RT			Cu @ RT			Obtained vol%
CGO: CuO	wt%	a (Å)	vol%	wt%	a (Å)	vol%	CGO: Cu
40:60	34.4(2)	5.4169(2)	39(1)	65.6(6)	3.6144(1)	61(1)	39:61
45:55	37.9(3)	5.4150(3)	43(1)	62.1(8)	3.6146(2)	57(3)	43:57
50:50	42.7(2)	5.4173(1)	48(1)	57.2(5)	3.61498(6)	52(2)	48:52
55:45	48.5(3)	5.4170(1)	54(2)	51.5(6)	3.61445(8)	46(2)	54:46
60:40	53.9(3)	5.4167(1)	59(1)	46.1(6)	3.6146(1)	41(2)	59:41
70:30	65.6(4)	5.4206(2)	70(1)	34.4(3)	3.61582(9)	30(1)	70:30

**Fig. 5:**
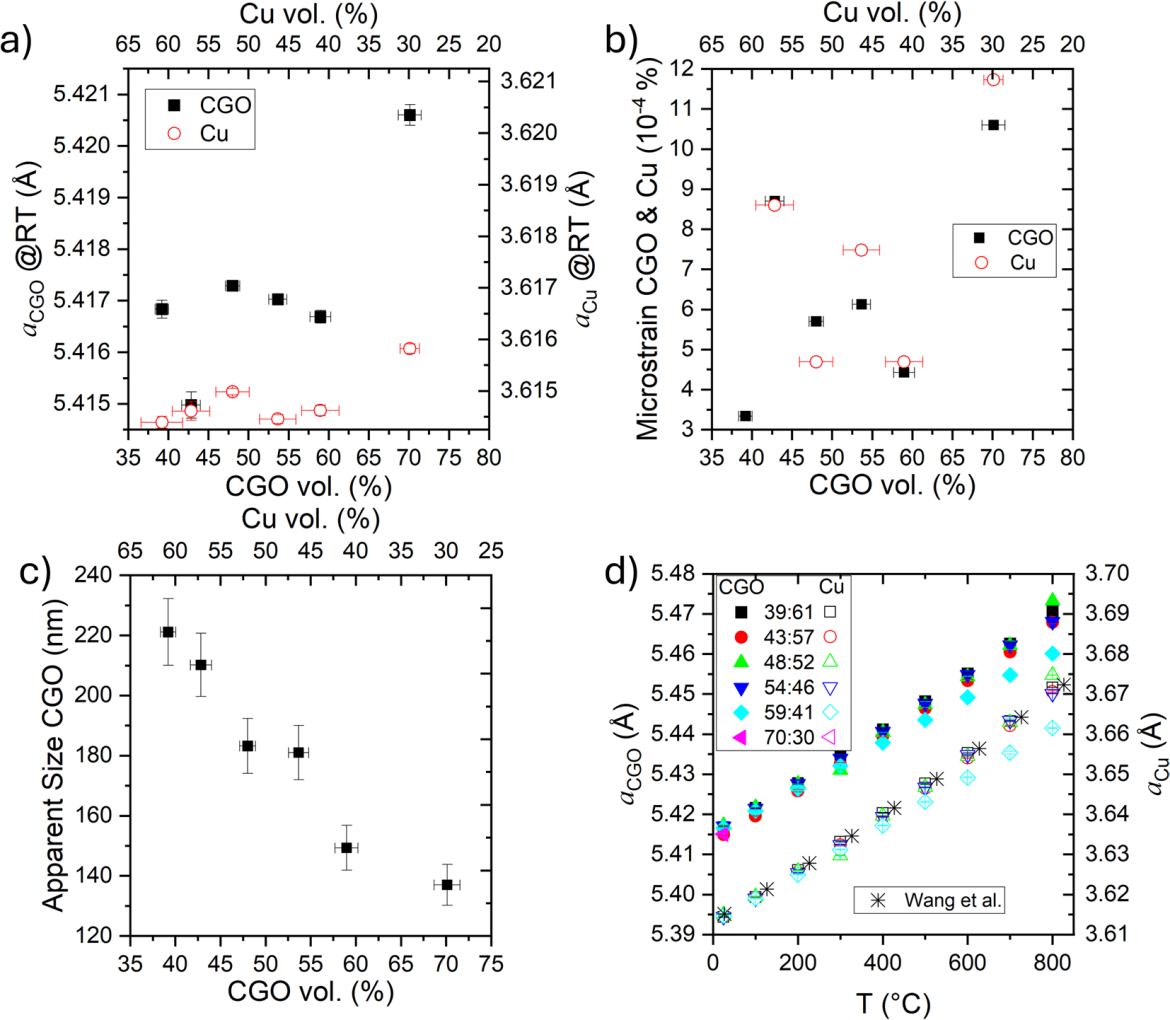
**a** Lattice parameter of CGO (left, black-solid symbols) and Cu (right, red-open symbols), **b** microstrain of CGO and Cu, and **c** crystallite size of CGO obtained from Rietveld refinement of XRD patterns at RT as a function of the vol% of CGO (bottom) and Cu (top); **d** Temperature evolution of CGO and Cu lattice parameters for the different composites and reported data^23^.

Figure 5a shows the RT lattice parameters of CGO and Cu obtained from Rietveld refinement of the XRD patterns as a function of CGO content. Both phases exhibit a slight increase in the lattice parameters with increasing CGO content, reaching a maximum at the highest CGO fraction (with increases of 0.068% for CGO and 0.039% for Cu). These small variations are consistent with residual elastic strain generated during the high-temperature synthesis and subsequent reduction processes, primarily due to the thermal expansion mismatch between the ceramic and metallic phases. The lattice parameter of CGO is significantly larger (5.42573 Å; ICSD 182976) than that of the metallic Cu (3.613 Å; ICSD 53247). As the CGO fraction increases, the mechanical constraint exerted by the Cu phase on the CGO lattice is progressively reduced, leading to a slightly larger CGO lattice parameter, while the Cu phase experiences increased interfacial constraint, resulting in lattice parameters marginally higher than those of stress-free Cu.

The broadening of the Bragg reflections, extracted from the Rietveld refinement, was analyzed using the Williamson–Hall size–strain approach, enabling the determination of the average microstrain and apparent crystallite size. The refined microstrain values of both CGO and Cu at RT (Fig. 5b) increase with increasing CGO content. This trend is consistent with the lattice parameter variations observed in Fig. 5a and supports their interpretation in terms of residual elastic strain induced by thermal expansion mismatch and interfacial constraint between the ceramic and metallic phases. As the CGO fraction increases, the interfacial area and mechanical constraint within the composite are enhanced, leading to an increase in the average microstrain in both phases, with the more compliant Cu phase accommodating a larger fraction of the elastic distortion. The non-monotonic evolution of the lattice parameters, suggests that additional microstructural factors, such as particle size distribution, phase connectivity, and local stress relaxation, also influence the measured average lattice parameters.

The apparent crystallite size of the CGO phase at RT (Fig. 5c) decreases with increasing CGO content, in agreement with the trend in SEM observations. Nevertheless, the crystallite sizes estimated from Rietveld refinement are approximately half the particle sizes measured by SEM, which is expected since XRD provides coherently diffracting domains, whereas SEM provides grain-scale information. Furthermore, the coarsening of CGO particles induced by the incorporation of metallic sintering aids has been extensively reported in the literature^18,19^. This growth is attributed to the reduction process at 800 °C for 10 h and becomes more pronounced at higher Cu fractions. The enhancement of CGO particle growth with increasing Cu content is likely associated with liquid phase assisted mass transport and modifications of the defect chemistry, which promote grain boundary mobility during reduction. The presence of Cu at sintering temperatures enhances grain boundary mobility through the formation of highly diffusive intergranular regions, while simultaneously altering the local defect structure of CGO, thereby reducing the energetic barrier for grain growth^20,21^. Additionally, the high thermal conductivity of Cu may further contribute to improved heat transfer during processing, thereby facilitating densification. Once the cermet was formed, no changes in peak broadening were observed as a function of temperature (as seen in Fig. 4b), indicating that no further CGO grain coarsening occurred in the studied temperature range. The Cu Bragg reflections were too sharp for the reliable extraction of the crystallite size. The Williamson–Hall size–strain approach is applicable only within a limited crystallite size range, typically from approximately 5–200 nm. For larger crystallite sizes, the diffraction peak broadening becomes negligible compared to instrumental broadening, making any size estimation inaccurate or meaningless^22^. In our samples, the Cu phase crystallites are well above this applicable range, as evidenced by the very sharp diffraction peaks observed in the XRD patterns.

The temperature-dependent evolution of the lattice parameters for both CGO and Cu, from RT to 800 °C is depicted in Fig. 5d. The different slopes of the curves anticipate variations in the thermal expansion coefficient (TEC) of each phase depending on the composite composition. The temperature dependence of the Cu lattice parameter aligns well with reported values^23^, except for the sample 59:41, which exhibits a noticeably lower slope.

**Fig. 6:**
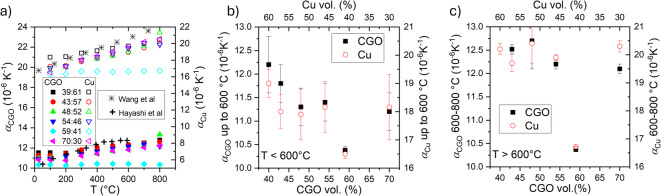
**a** Linear TEC, α, of the CGO (left axis) and Cu (right axis) individual phases as a function of the measured temperature for the different composites together with some reported data^23,31^, **b** average α values of CGO up to 600 °C, and **c** from 600 to 800 °C, as a function of the vol% of CGO (bottom) and vol% of Cu (top).

To highlight the differences in the TEC, Fig. 6a presents the calculated linear TEC, *α*_*XRD*_, of the CGO and Cu phases in the various cermet composites. These values (presented by solid and empty symbols, respectively) were calculated from the XRD data using Eq. (1). The average phase‑resolved linear TEC*s* up to 600 °C and from 600 to 800 °C are shown as a function of the cermet composition (vol% of each phase) in Fig. 6b and c, respectively. The results revealed that the cermet composition significantly influences the TEC*s* of both the CGO and Cu phases, with α_*XRD*_ decreasing as the CGO content increases, particularly in the lower temperature range. From the temperature evolution of α_XRD_ for the CGO and Cu phases across the full temperature range (RT to 800 °C, Fig. 6a), it can be seen that although the sample with 59:41 shows minimum and almost constant α_XRD_ values, the rest of compositions displayed the expected nonlinear increase in α with the temperature for both phases. To explain this increase, different factors must be considered. The TEC of CGO ranges from 10 × 10^− 6^ to 14 × 10^− 6^ K^− 1^ (typically 12.5 × 10^− 6^ for CGO between 50 and 1000 °C), and it increases nonlinearly with temperature. This nonlinearity is attributed to the asymmetric nature of the interatomic potential energy curve, as described by the lattice energy theory^24^. In addition, the TEC of CGO is known to increase linearly with the concentration of oxygen vacancies, regardless of whether these are introduced via Gd^3+^ doping or extrinsic reduction processes, owing to the associated decrease in binding energy^25^. For Cu, the TEC also exhibits a nonlinear increase with temperature, ^31^ typically ranging from 17 × 10^− 6^ to 25 × 10^− 6^ K^− 1^ from 25 °C to 600 °C, and it has been reported a maximum near 450 °C^26^. Besides, a decrease in the porosity of the samples, due to further densification of the composites in the 600–800 °C range, can explain the observed increase of the TEC of the samples at higher temperatures^27^. The anomalous behavior of the sample 59:41, which maintains almost constant *α*_*XRD*_ values for both phases throughout the entire temperature range, likely requires consideration of additional grain boundary or interfacial effects, beyond the known nonlinear dependence of TEC on temperature, to be fully explained.

**Fig. 7:**
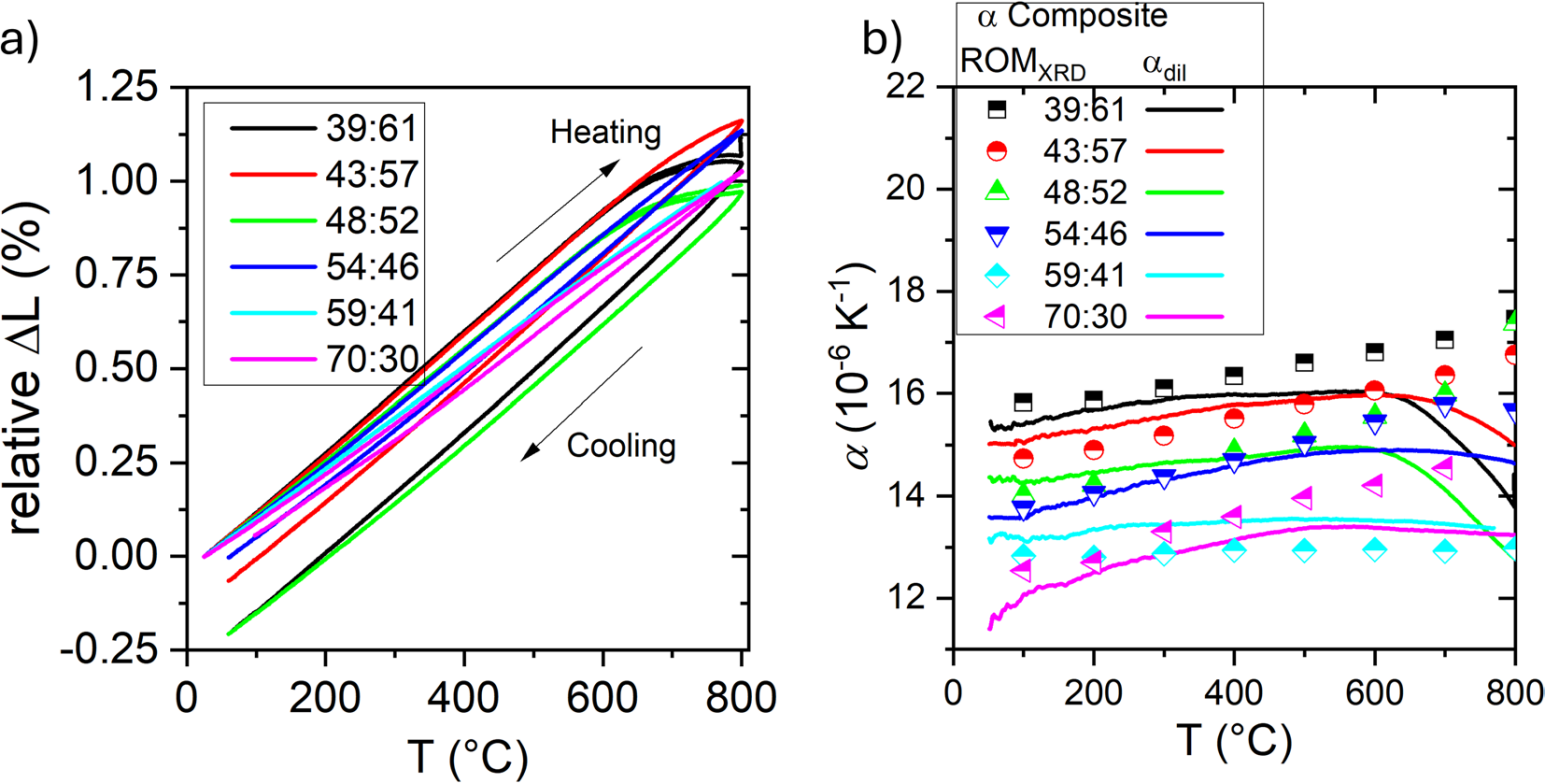
**a** Relative change in length (%) as a function of the temperature and **b** comparison of α of the different composites obtained from CGO and Cu and ROM model with XRD data (crossed symbols) and from dilatometer (lines) as a function of the temperature.

Simultaneous dilatometry recorded during in situ XRD diffraction measurements provides macroscopic expansion curves, *ΔL* (Eq. 2), as depicted in Fig. 7a for heating and cooling. At high temperatures (above 600 °C), the initial linear thermal expansion deviates downward, and upon cooling, a negative hysteresis is observed, indicating progressive sintering and shrinkage due to porosity reduction. The calculated bulk linear thermal expansion coefficients during heating (*α*_*dil*_, Eq. 3) for the different samples are shown in Fig. 7b (lines) (see average values in the low temperature and high temperature ranges in Supporting Figure S3). Figure 7b also shows the calculated thermal expansion coefficient of the cermet using the rule of mixtures (ROM) that is $\:{\alpha\:}_{c,\:ROM}={\alpha\:}_{1}{V}_{1}+{\alpha\:}_{2}{V}_{2}$, where $\:{\alpha\:}_{c,\:ROM}$, $\:{\alpha\:}_{1}$ and $\:{\alpha\:}_{2}$ are the TEC of the cermet, the phase 1 and phase 2, respectively, while $\:{V}_{1}\:$and $\:{V}_{2}$ are the volume fractions of phase 1 and phase 2, respectively.

Above ~ 600 °C, the different α_dil_ curves measured with the dilatometer cross, indicating that thermal expansion cannot be explained solely by the thermal expansion of the individual CGO and Cu phases. This behavior is attributed to post-sintering densification (Fig. 7a): Cu diffusion along grain boundaries, particle aggregation, and rearrangement reduce open porosity^28,29^, while increased oxygen-vacancy concentration in CGO promotes mass transport and local lattice relaxation^21,25^. As a result, samples with higher Cu content and higher initial porosity (Fig. 3b) exhibit a more pronounced decrease in α_dil_. Notably, the sample 59:41, shows an almost constant α_dil_ across the temperature range, correlating its volume change behavior and indicating minimal microstructural influence.

Up to 600 °C, the *α*_*dil*_ values follow the phase-resolved trend derived from the ROM_XRD_ data, obtained from Rietveld analysis. The slightly lower measured α_dil_values could be related to the porosity, as pores do not contribute to thermal expansion and act as compliant regions that relax internal stresses, while weakening load transfer between phases. Besides, it should be pointed out that the influence of porosity in TEC also depends on pore morphology and connectivity^30^. Typically, the ROM approximation assumes that there is no phase interaction between the constituent phases of a composite, thereby neglecting the effects of the microstructure and strain interactions, which significantly influence the thermal expansion behavior of the composites^23,31^. Differently from this ROM_XRD_ calculation, we considered the individual *α*_*XRD*_ values of each phase, as determined by in situ diffraction measurements, which inherently reflect microstructural interactions, primarily arising from the thermal expansion mismatch between Cu and CGO, but with stress-related porosity contributions.

To compare these data with theoretical models, we considered those that predict the TEC of two-phase materials, considering the difference in their shape (Kerner model) or elastic constants (Turner), which provide upper and lower bounds for the composite TEC, respectively^32^. The Turner model incorporates the influence of the elastic moduli in estimating the TEC of the composite. In this framework, the thermal stresses that induce strain in one phase are partially constrained by the stiffness of the other phase, making the mechanical properties of the individual components critical to the overall TEC. According to Turner’s assumptions, the dimensional change in each phase is restricted to match the average dimensional change of the composite, and shear deformation is neglected in the calculations. In this model, the TEC of the composite is given by:

$$\:{\alpha\:}_{c,\:Turner}=\:\frac{\left({\alpha\:}_{p}{V}_{p}{K}_{p}\right)+\left({\alpha\:}_{m}{V}_{m}{K}_{m}\right)}{{V}_{p}{K}_{p}+{V}_{m}{K}_{m}.}\:$$

where *α* is the TEC, *V* is the volume fraction, *K* is the bulk modulus, and subscripts *p*, *m* and *c* represent particle, matrix and composite, respectively^22^. The Kerner model assumes that ceramic particles are spherical and uniformly embedded within a continuous metallic matrix. Therefore, the TEC of the composite is considered equivalent to that of a representative volume element consisting of a spherical ceramic particle surrounded by a concentric shell of metallic matrix, with both phases occupying the same volume fractions as in the actual composite. This model provides the composite TEC as:

$$\:{\alpha\:}_{c,\:\mathrm{K}\mathrm{e}\mathrm{r}\mathrm{n}\mathrm{e}\mathrm{r}}={\alpha\:}_{p}{V}_{p}+{\alpha\:}_{m}{V}_{m}+{V}_{p}{V}_{m}\left({{\alpha\:}_{p}-\alpha\:}_{m}\right)\frac{{K}_{p}-{K}_{m}}{{V}_{m}{K}_{m}+{V}_{p}{K}_{p}+\left({3K}_{m}{K}_{p}/4{G}_{m}\right)}$$

where *G* is the shear modulus^33,34^.

**Fig. 8:**
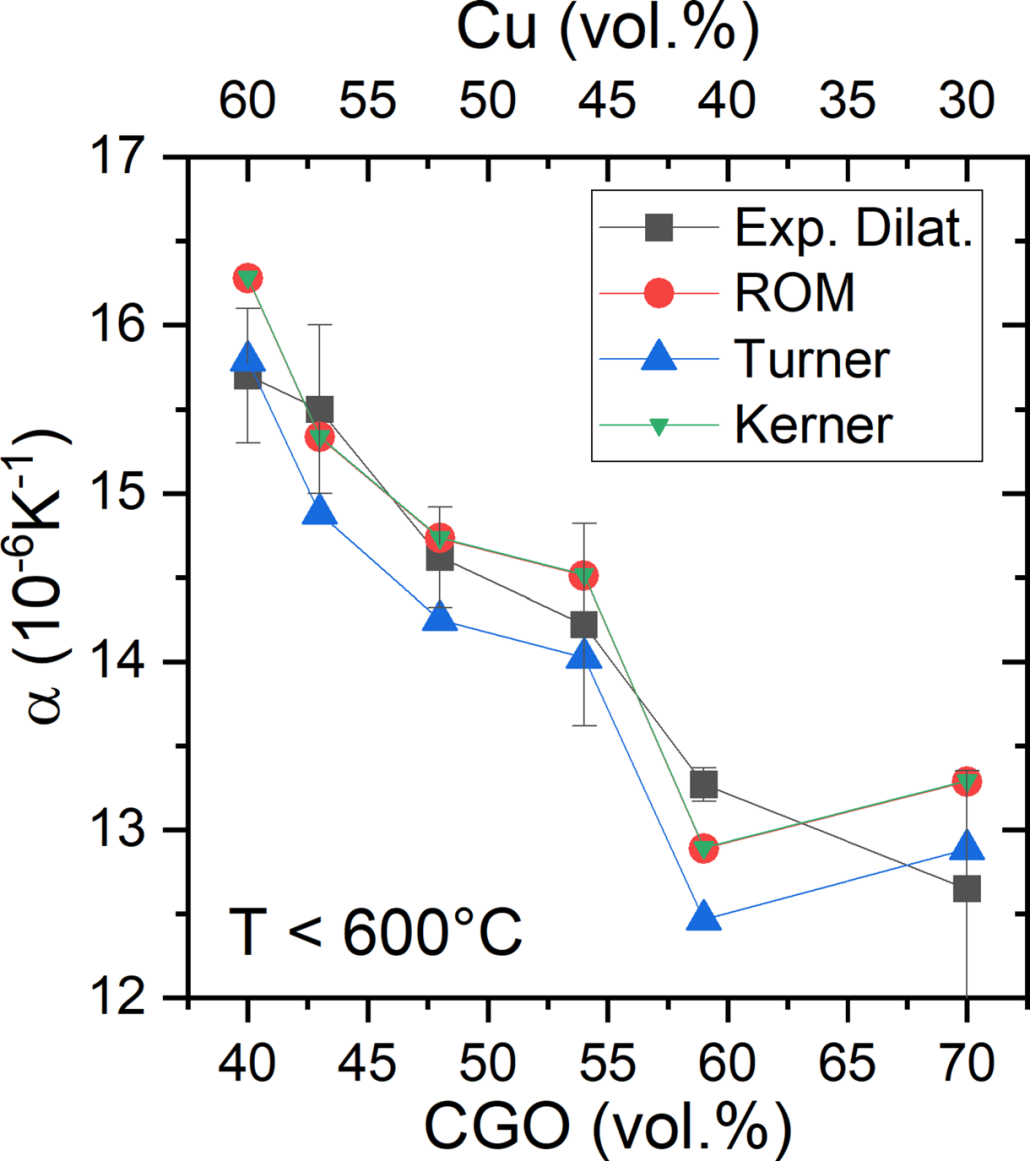
TEC of CGO–Cu composites obtained from dilatometer (average value up to 600 °C), calculated by ROM and XRD averaged data up to 600 °C and assuming different theoretical models that contemplate the difference in their elastic constants, Turner equation, and shape, Kerner.

Assuming *K*_CGO_=187.75 GPa, *K*_Cu_ = 140 GPa, and *G*_CGO_ = 80 GPa, *G*_Cu_ = 48 GPa^22,24–26^, and taking *α* values for CGO and Cu from XRD measurements at 100 °C (reference value), we can calculate *α* for the composites according to the different models. Figure 8 shows the average *α* values obtained from the dilatometer up to 600 °C (black squares, see Supporting Figure S3), calculated by ROM (red circles) from averaged XRD data up to 600 °C (Fig. 6b) and assuming two different theoretical models that account for the difference in their elastic constants (Turner, green up triangles) or shape (Kerner, blue down triangles). As expected, Turner provides the lowest values, and Kerner and ROM provide very similar results, since the third term in the Kerner equation can be neglected in this case. Therefore, no elastic or shape conclusions can be obtained when comparing the bulk and microstructural TEC measurements.

In summary, and in line with the electrode design criteria defined above, the measurements indicate that, from a thermomechanical perspective, the CGO–Cu 59:41 composite, exhibiting the lowest and most stable thermal expansion coefficient, is expected to provide mechanically stable contact with a CGO electrolyte.

## Conclusions

This study demonstrates that the thermal expansion behavior of CGO–Cu cermets is strongly influenced by their microstructural characteristics, particularly porosity, grain size, and phase distribution, which vary systematically with the CGO–Cu composition ratio (CGO: Cu). Through a combination of in situ synchrotron XRD and dilatometric measurements, it was revealed that both the CGO and Cu phases exhibit composition-dependent thermal expansion coefficients (TECs). Specifically, the 59:41 CGO–Cu sample was distinguished by an anomalously stable linear thermal expansion coefficient across the entire temperature range from room temperature to 800 °C. This unusual behavior correlates with a minimum in apparent CGO crystallite size and suggests the presence of strong interfacial or grain boundary effects that suppress further grain growth and dimensional changes, even at elevated temperatures.

Moreover, the experimental TEC values obtained macroscopically from dilatometry closely aligned with those predicted from the in situ XRD data using the rule of mixtures (ROM) model. This agreement indicates that the phase-specific TECs extracted via synchrotron diffraction already embed the effects of microstructural interactions such as thermal mismatch stress and grain-scale constraints. The deviation from the expected TEC trend observed above 600 °C in most samples, except for 59:41, is attributed to additional sintering and densification phenomena induced by the solid-state wetting effect of Cu at high temperatures. Thus, the 59:41 composite emerges as a particularly thermally stable configuration, offering potential for high-temperature applications in which dimensional stability is critical.

## Supplementary Information

Below is the link to the electronic supplementary material.

Supplementary Material 1

## Data Availability

Data would be available on request.

## Declarations

### Generative AI and AI-assisted technologies in the manuscript preparation process

During the preparation of this work, the authors used Microsoft Copilot in order to improve the readability and language of the work. After using this tool, the authors reviewed and edited the content as needed and take full responsibility for the content of the published article.
