# Correction: In situ high temperature X-ray diffraction and dilatometric analysis of CGO–Cu composites for solid oxide devices

**DOI:** 10.1038/s41598-026-37894-0

**Published:** 2026-02-05

**Authors:** M. Balaguer, M. Fabuel, A. Kriele, A. Stark, J. M. Serra, C. Solís

**Affiliations:** 1 Instituto de Tecnología Química (ITQ), Consejo Superior de Investigaciones Científicas-Universitat Politècnica de València, 46022 Valencia, Spain; 2 German Engineering Materials Science Centre (GEMS) at Heinz Maier-Leibnitz Zentrum (MLZ), Helmholtz-Zentrum Hereon, 85748 Garching, Germany; 3 Institute of Materials Physics, Helmholtz-Zentrum Hereon, Max-Planck-Strasse 1, 21502 Geesthacht, Germany

Correction to: *Scientific Reports* 10.1038/s41598-026-35161-w, published online 10 January 2026

The original version of the Article contained an error in Fig. 6 c). Due to an error during the figure assembly, Fig. 6 c) was a duplication of Fig. 6 b).

The original Fig. 6 and accompanying legend appear below.

**Fig. 6:**
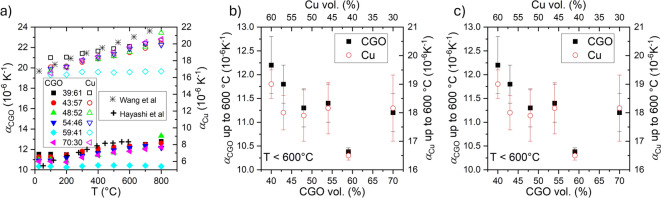
(**a**) Linear TEC, α, of the CGO (left axis) and Cu (right axis) individual phases as a function of the measured temperature for the different composites together with some reported data^23,31^, (**b**) average α values of CGO up to 600 °C, and (**c**) from 600 to 800 °C, as a function of the vol% of CGO (bottom) and vol% of Cu (top).

The original Article has been corrected.

