# Supplementary material for: In situ high temperature X-ray diffraction and dilatometric analysis of CGO–Cu composites for solid oxide devices
Source: Sci Rep. 2026 Jan 10;16:1315. doi: 10.1038/s41598-026-35161-w (PMC12796496; doi:10.1038/s41598-026-35161-w)
Supplement: Supplementary file 1 — Supplementary Material 1 [file 41598_2026_35161_MOESM1_ESM.docx]

**Supporting Information**

**Supporting Figure S1**


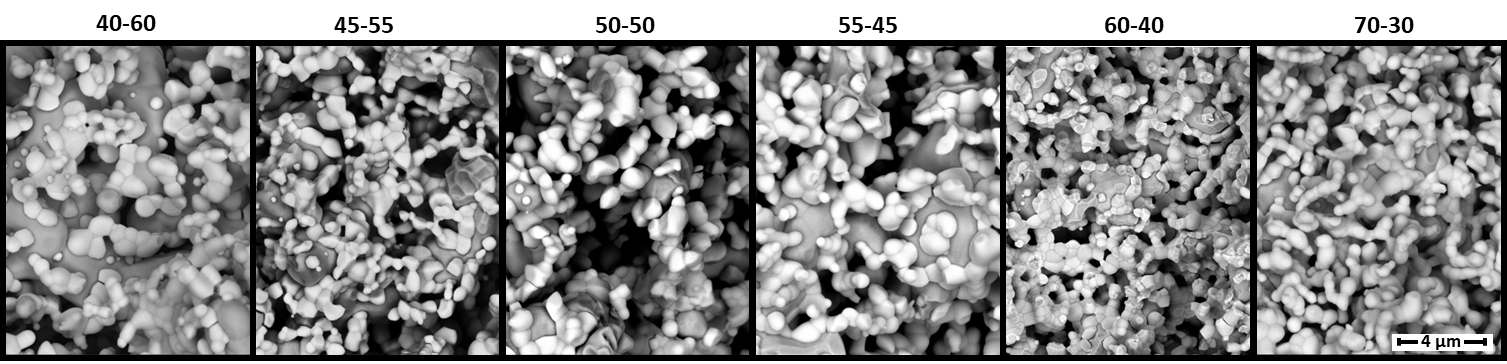


Supporting Figure S1: SEM images used for grain size determination plotted in Figure 1b

**Supporting Figure S2**

**
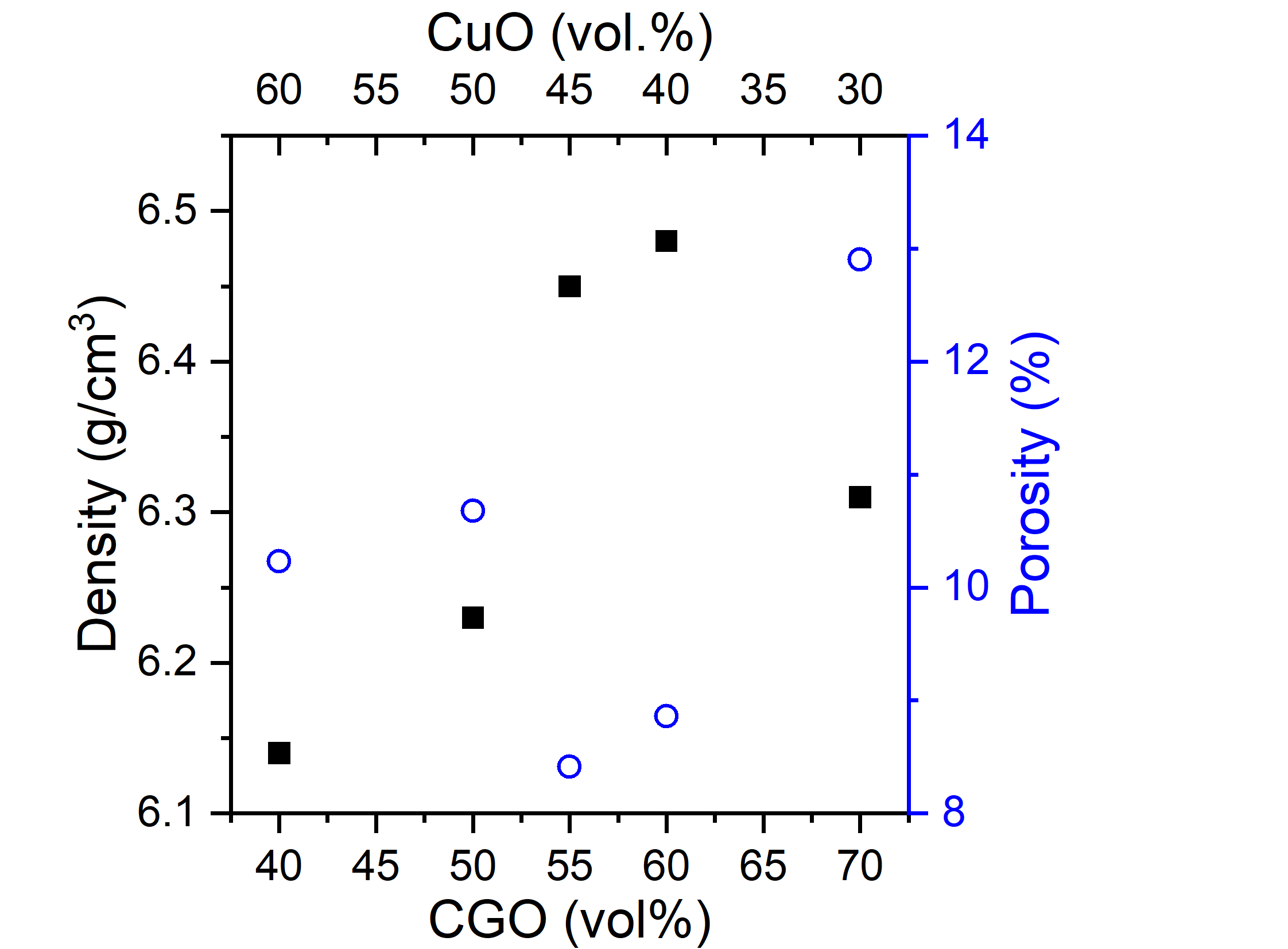
**

Supporting Figure S2: density (left axis) and porosity (right axis) obtained from Archimedes method of the samples before reduction.

Porosity values of the CGO-CuO as prepared composites are much lower than the CGO – C reduced specimens. This is due to the fact that thee reduction of dense CuO to metallic Cu is accompanied by a large theoretical volume contraction (~44%), which inevitably leads to the formation of significant porosity. As a result, even nearly dense CuO bodies can develop porosity levels on the order of 30 - 45% after reduction, depending on temperature and reduction kinetics**.**

**Supporting Figure S3**

**
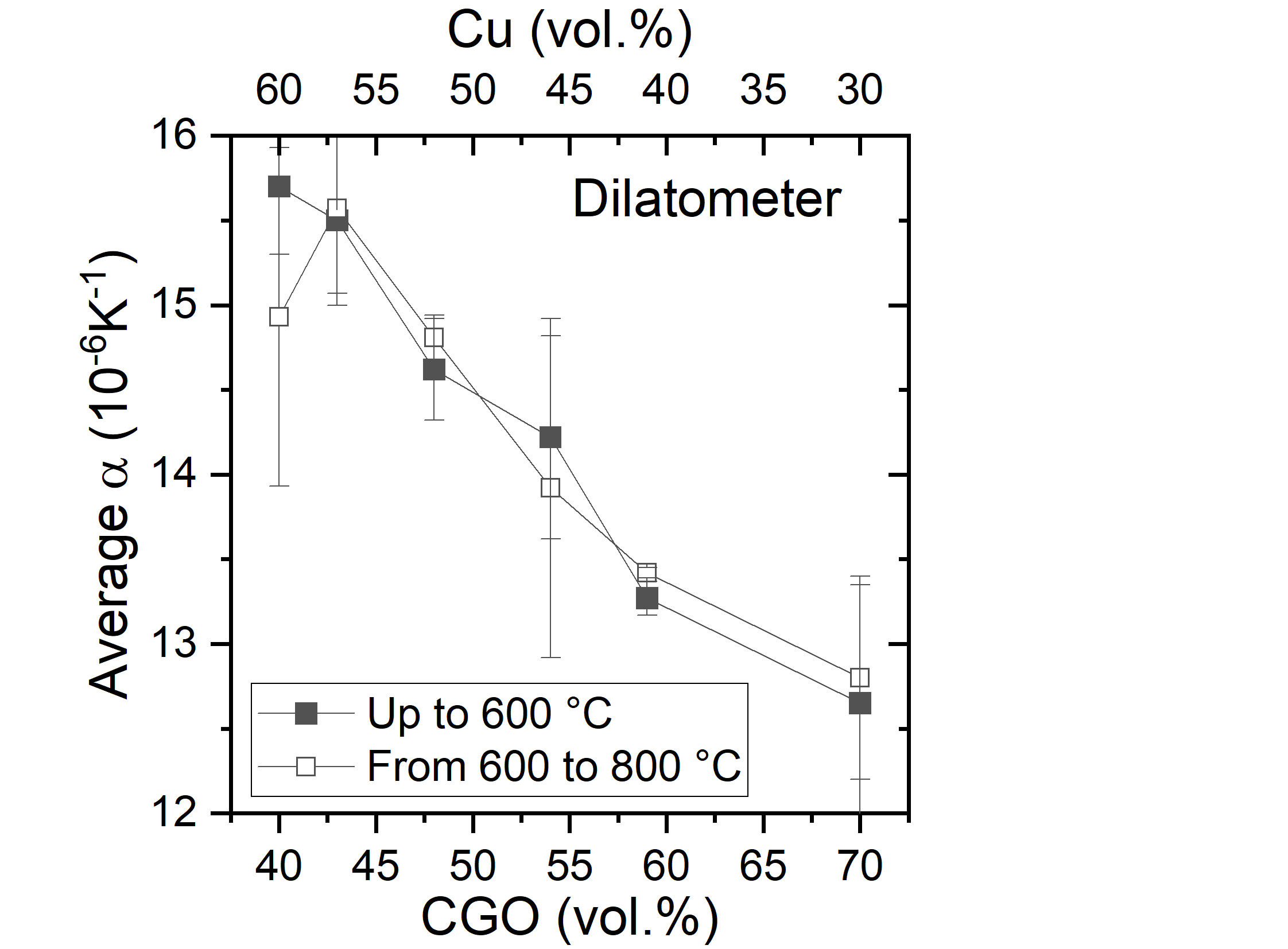
**

Supporting Figure S3: Average α values obtained from dilatometer curves up to and from 600 °C
